# Quality assessment of case reports in high‐impact urology journals using SCARE guideline

**DOI:** 10.1002/hsr2.353

**Published:** 2021-08-05

**Authors:** Asaad Moradi, Mehran Moghimian, Alireza Ghoreifi, Behnam Shakiba

**Affiliations:** ^1^ Department of Urology, Firoozgar Hospital Iran University of Medical Sciences Tehran Iran; ^2^ Department of Urology, Hasheminejad Hospital Iran University of Medical Sciences Tehran Iran; ^3^ Institute of Urology, Norris Comprehensive Cancer Center University of Southern California Los Angeles California USA

**Keywords:** case report, journal, quality, urology

## Abstract

**Background and Aims:**

The usefulness of case reports is dependent on the complete, consistent, and rigorous reporting of these cases. In order to provide a standard guideline for reporting surgical case reports, the SCARE (Surgical CAse REport) guidelines were developed in 2016. The present study evaluated the completeness and transparency of published case reports in high‐impact urology journals based on the SCARE guideline.

**Methods:**

This cross‐sectional study was performed on 100 case reports published in Urology, Urology Journal, BMC Urology, and Urology Case Reports journal. Two independent reviewers performed the scoring using the last version of SCARE statement. Each of the 34 items of SCARE guideline were classified as “yes” if the item was reported in the case report text. The SCARE items were classified as “no” when the authors of case reports had not reported that item or could not tell something about reporting the item. Completeness of reporting (COR) score was calculated for each case report. COR score (%) is defined as [“yes” answers/(“yes” answers + “no” answers)] × 100 for each case report.

**Results:**

The mean COR score for all the assessed case reports was 49%, ranging from 21% to 79%. Topics with the highest mean COR score were introduction (77% ± 42%), additional information (75% ± 43%), patient information (65% ± 19%), and abstract (66% ± 24%). In contrast, topics with the lowest mean COR were patient perspective (1% ± 10%) and keywords (3% ± 17%).

**Conclusion:**

The present study showed that case reports published in urology journals suffer from insufficient reporting. SCARE or CARE guidelines can provide a framework for assessing the reporting quality of case reports before publication. Nevertheless, further studies are highly recommended to better evaluate the efficacy of these guidelines' endorsement on the quality of case reports published in urology journals.

## 1 INTRODUCTION

In general, case reports have been formally placed at the lowest level in the hierarchy of evidence.^1^ However, they still have a profound role in the discovery of new diseases, advancements made in surgical interventions, and medical education.^2^, ^3^ In recent years, the number of case reports published in medical journals has significantly increased and over 2 million case reports are currently recorded in PubMed indexed journals.^4^ Urology is no exception to this fact and such case reports have an important role in introducing new diseases and their natural history, introducing rare diseases or rare presentations of a disease, investigation and treatment of rare diseases, as well as reporting unique surgical complications and their management.^2^, ^5^ Moreover, a growing number of case reports in urology journals have led to the creation and launch of several international, peer‐reviewed journals that explicitly publish case reports, for example, Urology Case Reports, International Journal of Urology (IJU) Case Reports, Case Reports in Urology, and Journal of Endourology Case Reports.

The usefulness of case reports is dependent on the complete, consistent, and rigorous reporting of these cases. Case reports written without considering the reporting standards are not fully approved to guide clinical practice or to assist in clinical study design.^6^ Improved reporting, a complete description of clinical details, and explicit conclusions may lead to reduced “waste in research.”^7^ In order to provide a standard guideline for reporting case reports, the CARE (CAse REport) guidelines were developed in 2013 using the Delphi method.^6^ CARE was the first guideline that was developed with the aim of improving the completeness, transparency, and quality of case reports.^8^ Some researchers claimed that CARE is not surgically focused; hence, they developed the SCARE guideline by the Delphi expert consensus method to further improve the reporting quality of surgical case reports. The SCARE statements were developed in 2016 and updated in 2018.^5^, ^9^

To the authors' knowledge, only a limited number of studies to date have evaluated the completeness and quality of case reports.^8^, ^10^, ^11^, ^12^ No study has yet focused on assessing the quality and transparency of reporting case reports in the field of Urology. Therefore, we conducted this study with the aim of evaluating the completeness and transparency of published case reports in high‐impact urology journals based on the SCARE guideline.

## 2 MATERIAL AND METHODS

### 2.1 Selection of eligible case reports

This cross‐sectional study was performed on case reports published in three urology journals (Urology, Urology Journal, and BMC Urology) from May 1, 2019 to May 1, 2020. These journals publish case reports along with other types of articles and have a higher impact factor among the various journals in the field of Urology. The journals' impact factors were obtained from the 2019 edition of the Science Citation Index Journal Citation Reports. Case reports that reported more than one patient were excluded from the present study. Two authors reviewed all issues of these journals in the mentioned study period for case reports. After the identification of all case reports, 50 case reports were randomly included using the random numbers generator. In addition, we also included 50 random case reports from the “Urology Case Reports” journal in the same time period. This journal has the highest impact factor among urology journals that only publish case reports.

### 2.2 Data extraction

We used the method of Agha et al^5^ for data extraction as a valid method. In brief, two independent reviewers (M.M. and A.G.) performed the scoring using the last version of SCARE statement.^9^ Each of the 34 items of SCARE guideline (Table 1) were classified as “yes” if the item was reported in the case report text. The SCARE items were classified as “no” when the authors of case reports had not reported that item or could not tell something about reporting the item. Disagreement between the reviewers was dealt through consensus with a third researcher (B.S.).

**TABLE 1:** SCARE Guideline 2018

SCARE 2018 checklist		
Topic	Item	Checklist item description
Title	1	The words “case report” should appear in the title. The title should also describe the area of focus (eg, presentation, diagnosis, surgical technique or device or outcome)
Keywords	2	Three to six key words that identify areas covered in this case report (include “case report” as one of the keywords)
Abstract	3a	Introduction—Describe what is unique or educational about the case (ie, what does this work add to the surgical literature, and why is this important?)
	3b	Presenting complaint and investigations—describe the patient's main concerns and important clinical findings
	3c	The main diagnoses, therapeutics interventions, and outcomes
	3d	Conclusion—Describe the main lessons to “take‐away” from this case study
Introduction	4	Background—summarise what is unique or educational about the case. Give reference to the relevant surgical literature and current standard of care. The background should be referenced, and one to two paragraphs in length
Patient information	5a	Demographic details—include deidentified demographic details on patient age, sex, ethnicity, occupation. Where possible, include other useful pertinent information, for example, body mass index and hand dominance
	5b	Presentation—describe the patient's presenting complaint (symptoms). Describe the patient's mode of presentation (brought in by ambulance or walked into Emergency room or referred by family physician)
	5c	Past medical and surgical history, and relevant outcomes from interventions
	5d	Other histories—Describe the patient's pharmacological history including allergies, psychosocial history Drug, Smoking, and if relevant, accommodation, walking aids), family history including relevant genetic information
Clinical findings	6	Describe the relevant physical examination and other significant clinical findings. Include clinical photographs where relevant and where consent has been given
Timeline	7	Inclusion of data which allows readers to establish the sequence and order of events in the patient's history and presentation (using a table or figure if this helps). Delay from presentation to intervention should be reported
Diagnostic assessment	8a	Diagnostic methods—describe all investigations taken to arrive at methods: physical exam, laboratory testing, radiological imaging, and histopathology
	8b	Diagnostic challenges—describe what was challenging about the diagnoses, where applicable, for example, access, Financial, cultural
	8c	Diagnostic reasoning—Describe the differential diagnoses and why they were considered
	8d	Prognostic characteristics when applicable (eg, tumor staging or for certain genetic conditions). Include relevant radiological or histopathological images in this section
Therapeutic intervention	9a	Pre‐intervention considerations—if there were patient‐specific optimisation measures taken prior to surgery or other intervention these should be included, for example, treating hypothermia/hypovolaemia/hypotension in a burns patient, Intensive care unit treatment for sepsis, dealing with anticoagulation/other medications, and so on
	9b	Interventions—describe the type(s) of intervention(s) deployed (pharmacologic, surgical, physiotherapy, psychological, preventive). Describe the reasoning behind this treatment offered. Describe any concurrent treatments (antibiotics, analgesia, anti‐emetics, nil by mouth, Venous thromboembolism, Prophylaxis, etc.). Medical devices should have manufacturer and model specifically mentioned
	9c	Intervention details—describe what was done and how. For surgery include details on: anesthesia, patient position, use of tourniquet and other relevant equipment, prep used, sutures, devices, surgical stage (one or two stage, etc.). For pharmacological therapies include information on the formulation, dosage, strength, route, duration, etc. Include intra‐operative photographs and/or video or relevant histopathology in this section. Degree of novelty for a surgical technique/device should be mentioned, for example, “first in human”
	9d	Who performed the procedure—operator experience (position on the learning curve for the technique if established, specialisation and prior relevant training). For example, “junior resident with 3 years of specialised training”
	9e	Changes—if there were any changes in the interventions, describe these details with the rationale
Follow‐up and outcomes	10a	Follow‐up—describe When the patients was followed up Where How (imaging, tests, scans, clinical examination, phone call), and Whether there were any specific postoperative instructions. Future surveillance requirements—for example, imaging surveillance of endovascular aneurysm repair or clinical exam/ultrasound of regional lymph nodes for skin cancer
	10b	Outcomes—Clinician assessed and (when appropriate) patient reported outcomes (eg, questionnaire details). Relevant photographs/radiological images should be provided, for example, 12 month follow‐up
	10c	Intervention adherence/compliance—where relevant how well patient adhered to and tolerated their treatment. For example, post‐operative advice (heavy lifting for abdominal surgery) or tolerance of chemotherapy and pharmacological agents
	10d	Complications and adverse events—all complications and adverse or unanticipated events should be described in detail and ideally categorised in accordance with the Clavien‐Dindo Classification. How they were prevented, diagnosed and managed. Blood loss, operative time, wound complications, reexploration/revision surgery, 30‐day post‐op and long‐term Morbidity/mortality may need to be specified. If there were no complications or adverse outcomes this should also be included
Discussion	11a	Strengths—describes the strengths of this case
	11b	Weaknesses and limitations in your approach to this case. For new techniques or implants contraindications and Alternatives, potential risks and possible complications if applied to a larger population. If relevant, has the case been reported to the relevant national agency or pharmaceutical company (eg, an adverse reaction to a device)
	11c	Discussion of the relevant literature, implications for clinical practice guidelines and any relevant hypothesis generation
	11d	The rationale for your conclusions
	11e	The primary “take‐away” lessons from this case report
Patient perspective	12	When appropriate the patient should share their perspective on the treatments they received
Informed consent	13	Did the patient give informed consent for publication? Please provide if requested by the journal/editor. If not given by the patient, explain why, for example, death of patient and consent provided by next of kin or if patient/family untraceable then document efforts to trace them and who within the hospital is acting as a guarantor of the case report
Additional information	14	Conflicts of Interest, sources of funding, institutional review board or ethical committee approval where required

### 2.3 Statistical analysis

The data were analyzed with the Statistical Package of Social Sciences (SPSS, Chicago, Illinois) for Windows version 19. A *P*‐value <.05 was considered as statistically significant. In this study, three types of analyses were performed:

First, calculation of completeness of reporting (COR) score for each case report: COR score (%) is defined as [“yes” answers/(“yes” answers + “no” answers)] × 100 for each case report.^5^ Of note, previous studies have shown that the COR score is an acceptable estimation for the analysis.^8^, ^13^

Second, descriptive analysis: the number and proportion of case reports reporting each of the SCARE items.

Thirdly, comparison of the COR score between journals that publish case reports only and journals that publish different types of articles using independent sample T‐test.

### 2.4 Ethical consideration

Although this study is a non‐risk observational research, the study protocol was approved by the Ethics Committee of Iran University of Medical Sciences (ethical approval code: IR.IUMS.REC.1399.670).

## 3 RESULTS

A total of 100 case reports were included in the present study. Of these, 50 articles were published in the Urology Case Reports journal, 19 in Urology (the Gold Journal), 11 case reports were published in the Urology journal, and 20 in BMC Urology journal.

**FIGURE 1:**
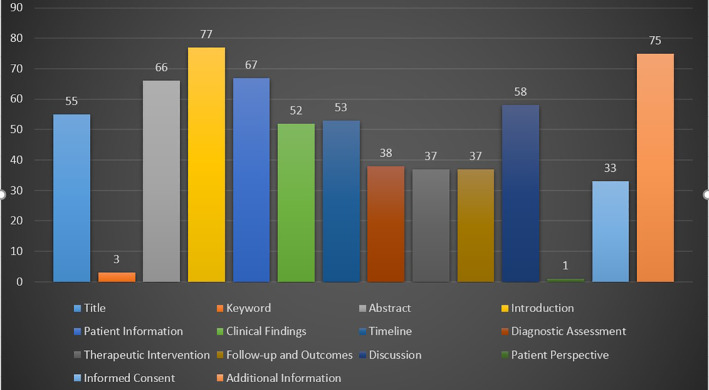
The mean completeness of reporting (COR) score for all the studied case reports

The mean COR score for all the assessed case reports was 49%, ranging from 21% to 79%. Topics with the highest mean COR score were introduction (77% ± 42%), additional information (75% ± 43%), patient information (65% ± 19%), and abstract (66% ± 24%). In contrast, topics with the lowest mean COR were patient perspective (1% ± 10%) and keywords (3% ± 17%; Figure 1).

The mean ± *SD* COR score was 52% ± 11% for case reports published in journals that publish different article types compared to 45% ± 7% for case reports published in “Urology Case Reports,” as the urology journal that publishes case reports only (*P* = .0001). Table 2 summarizes the mean COR scores for each topic of the SCARE statement in the studied case reports.

**TABLE 2:** Compliance with the SCARE items in journals that publish different article types and journal which publishes case reports only

SCARE items	Compliance with SCARE item in journal which publishes case reports only	Compliance with SCARE item in journals which publish different article types	Compliance with SCARE item in all journals
1	50%	60%	55%
2	0%	6%	3%
3a	76%	76%	76%
3b	60%	66%	63%
3c	88%	76%	82%
3d	36%	52%	44%
4	74%	80%	77%
5a	94%	100%	97%
5b	96%	90%	93%
5c	38%	64%	51%
5d	28%	26%	27%
6	50%	54%	52%
7	42%	64%	53%
8a	94%	92%	93%
8b	2%	8%	5%
8c	20%	30%	25%
8d	22%	36%	29%
9a	12%	18%	15%
9b	100%	98%	99%
9c	34%	56%	45%
9d	2%	2%	2%
9e	24%	26%	25%
10a	46%	64%	55%
10b	52%	54%	53%
10c	6%	4%	5%
10d	38%	30%	34%
11a	10%	28%	19%
11b	6%	22%	14%
11c	92%	100%	96%
11d	64%	80%	72%
11e	92%	86%	89%
12	0%	2%	1%
13	24%	42%	33%
14	68%	82%	75%

*Note*: The items were described in Table 1.

## 4 DISCUSSION

Some studies have confirmed that the validity and reliability of published articles are negatively affected by incomplete and poor reporting.^7^ This issue is a major problem as at least 50% of research papers have been regarded as waste due to insufficient reporting.^14^ This amount of unusable research products means a waste of time, energy, and resources. The present study shows that the overall completeness of reporting case reports in prestigious urology journals is low. Although case reports have a low level of evidence in the evidence hierarchy, yet in certain situations, case reports and case series may play an important role in subsequent clinical research. Therefore, case reports have the potential to attract a vast majority of readers.^15^, ^16^ None of the assessed journals in the present study had endorsed the SCARE or CARE statements in their “instructions for authors” section. Previous studies have confirmed that the explicit implementation of the SCARE or CARE guidelines in the mentioned section of journals can lead to improved reporting quality of surgical and non‐surgical case reports.^5^, ^8^ Therefore, we believe that now is the time to implement adherence to the SCARE statements in the “instructions for authors” section of urology journals. Adherence to the SCARE reporting guidelines can surely improve the quality of surgical case reports and increase the usefulness of these papers.

In the present study, “introduction,” “additional information,” “patient information,” and “abstract” were the most frequent items which were properly presented among the published case reports in high‐quality urology journals. In contrast, “patient perspective” and “keywords” were reported in a small proportion of the studied case reports. Based on the SCARE statement, “patient perspective” is defined as “the patients should share their perspective on the treatments they received.” The low COR score for “patient perspective” is expectable since authors and reviewers are not usually familiar with this aspect in reporting a case report. To our surprise, the item “keywords” had the second lowest percentage among the different subordinate items in published case reports. Low mean COR score for “keywords” is spectacular as in all the included journals the instructions for authors section recommends authors to provide some keywords in their manuscript. However, the reason for the low mean COR score for “keywords” is mainly related to the definition of keywords in the SCARE statement. SCARE recommends authors and journals to include the “case report” phrase as one of the keywords. Most case reports in our study provided three to five keywords but did not include “case report” as one of them.

Interestingly, in the current study, the mean COR score was significantly higher for case reports published in journals that publish different article types in comparison to “Urology Case Report” journal, which publishes case reports only. None of these two journal groups had encouraged authors to follow CARE or SCARE statements before manuscript submission.

Over the recent decade, several new peer‐reviewed journals have emerged focused on publishing case reports only. There is much debate on the value of these journals and some researchers believe that some case report journals are not reputable enough.^16^ Although these journals still need to strongly improve their quality, all authors are also expected to learn how to distinguish between reputable and non‐reputable journals.

The present study is one of the few studies that has assessed the quality of reporting case reports using the SCARE statement and the first study to evaluate high‐impact urology journals; yet it still has a number of limitations that must be acknowledged. First, we limited our investigation to case reports published in three urology journals and one journal publishing case reports only. Therefore, our results cannot be generalized to all urology journals. Second, all assessments were done on case reports published in high‐impact urology journals. Evaluation of higher impact urology journals may lead to higher quality scores compared to those from other urology journals. Third, we used SCARE 2018 as the last version of this statement at the time of study. During the review process of our manuscript, the new version of SCARE statement was published (December 2020),^17^ so it was not feasible for us to redo the study.

## 5 CONCLUSIONS

The present study confirms that case reports published in urology journals suffer from insufficient and poor reporting quality. SCARE or CARE guidelines can provide a framework for authors, reviewers, and editors to assess the reporting quality of case reports before publication. Nevertheless, further studies are recommended to better evaluate the efficacy of these guidelines' endorsement on the quality of case reports published in urology journals.

## CONFLICT OF INTEREST

The authors declare no conflict of interest in this study.

## AUTHOR CONTRIBUTIONS

Conceptualization: Behnam Shakiba

Data Curation: Alireza Ghoreifi, Mehran Moghimian, Asaad Moradi

Methodology: Mehran Moghimian, Asaad Moradi

Supervision: Behnam Shakiba

Writing—Original Draft Preparation: Asaad Moradi, Mehran Moghimian

Writing—Review & Editing: Behnam Shakiba, Alireza Ghoreifi

All authors have read and approved the final version of the manuscript.

Behnam Shakiba had full access to all of the data in this study and takes complete responsibility for the integrity of the data and the accuracy of the data analysis.

## TRANSPARENCY STATEMENT

Behnam Shakiba affirms that this manuscript is an honest, accurate, and transparent account of the study being reported; that no important aspects of the study have been omitted; and that any discrepancies from the study as planned (and, if relevant, registered) have been explained.

## Data Availability

The authors confirm that the data supporting the findings of this study are available within the article.
